# The University Magazine

**Published:** 1888-05

**Authors:** 


### Book Notices.

"The University Magazine" is the name of a publication, the prospectus for which has been issued by the indefatigable Dr. Hummel, of Philadelphia, publisher of several sterling periodicals.

It is to be a handsome sixty-four page monthly, and will appear October 1st. It will be edited under the auspices of the *Alumni* and Faculty of Medicine of the University of Pennsylvania, by a staff of able and well known physicians. Subscription price, $2 a year. Look out for it; it will be something out of the ordinary, and will create a sensation. Success to it and to its enterprising publisher.

